# In vitro biocompatibility of biohybrid polymers membrane evaluated in human gingival fibroblasts

**DOI:** 10.1002/jbm.b.34591

**Published:** 2020-02-25

**Authors:** Bin Guo, Chuhua Tang, Mingguo Wang, Zhongqi Zhao, Hassan A. Shokoohi‐Tabrizi, Bin Shi, Oleh Andrukhov, Xiaohui Rausch‐Fan

**Affiliations:** ^1^ Department of Stomatology Jinan Central Hospital affiliated to Shandong University Jinan Shandong China; ^2^ Division of Periodontology and Conservative Dentistry University Clinic of Dentistry, Medical University of Vienna Vienna Austria; ^3^ Department of Stomatology PLA Strategic Support Force Characteristic Medical Center Beijing China; ^4^ Department of Oral and Maxillofacial Surgery The First Affiliated Hospital of Fujian Medical University Fuzhou Fujian China

**Keywords:** adhesion, biohybrid polymers membrane, differentiation, extracellular matrix, gingival fibroblasts, migration, proliferation

## Abstract

The biohybrid polymer membrane (BHM) is a new biomaterial designed for the treatment of soft periodontal tissue defects. We aimed to evaluate the in vitro biocompatibility of the membrane in human gingival fibroblasts and the capability to induce cell adhesion, migration, differentiation and improving the production of the extracellular matrix. BHM and Mucograft® collagen matrix (MCM) membranes were punched into 6 mm diameter round discs and placed in 96‐well plates. Human primary gingival fibroblasts were seeded on the membranes or tissue culture plastic (TCP) serving as the control. Cell proliferation/viability and morphology were evaluated after 3, 7, and 14 days of culture by cell counting kit (CCK)‐8 assay and scanning electron microscopy, respectively. Additionally, the gene expression of transforming growth factor (TGF)‐β1, focal adhesion kinase (FAK), collagen type 1 (Col1), alpha‐smooth muscle actin (α‐SMA), and fibroblasts growth factor (FGF)‐2 was analyzed at 3, 7, and 14 days of culture by qPCR. Cell proliferation on BHM was significantly higher than on MCM and similar to TCP. Gene expression of TGF‐β1, FAK, Col1, and α‐SMA were significantly increased on BHM compared to TCP at most investigated time points. However, the gene expression of FGF‐2 was significantly decreased on BHM at Day 7 and recovered at Day 14 to the levels similar to TCP. The finding of this study showed that BHM is superior for gingival fibroblasts in terms of adhesion, proliferation, and gene expression, suggesting that this membrane may promote the healing of soft periodontal tissue.

## 1 INTRODUCTION

Regeneration of periodontal soft tissue is one of the main challenges of contemporary periodontology and implantology (Larsson et al., 2016). Conservative periodontal therapy alone is often insufficient for soft tissue recovery. To date, soft tissue augmentation using autologous tissue grafts is the first choice of use in periodontal surgery (Bertl, Melchard, Pandis, Muller‐Kern, & Stavropoulos, 2017; Zuhr, Baumer, & Hurzeler, 2014). Despite the good clinical outcome, autologous tissue grafts have some obvious disadvantages, such as the limited size, the necessity for the additional surgical site, pain to patients, damage risk of the palatal artery, and the differences in texture and color with adjacent tissues (Pietruska, Skurska, Podlewski, Milewski, & Pietruski, 2019). Therefore, researchers and clinicians have been exploring alternative tissue renewing materials “outside the palate”, and periodontal gingival surgery using xenogeneic materials or synthetic materials instead of autologous tissue has gradually developed (Vaquette et al., 2018).

Modern periodontal tissue regeneration is inconceivable without the application of different biomaterials like scaffolds or barrier membranes. The principles underlying the application of biological barrier membranes are the physical separation of different tissue compartments (Sheikh et al., 2017). Such separation allows optimizing the healing process of different tissues with distinct properties and minimizing the disturbing effects of neighboring tissues. The barrier membranes could be either nonresorbable or resorbable. The advantage of the resorbable membrane is that they are degrading with time, which allows reconstruction of natural tissue structures.

The requirements of clinicians for the barrier membrane became more rigorous within the last years. Modern membranes are expected not only to perform a barrier function but also to stimulate the natural process of wound healing. This can be achieved by modification of different properties such as the elasticity, three‐dimensional (3D) structure of the membrane and incorporation of various bioactive molecules (Chen et al., 2018; Omar, Elgali, Dahlin, & Thomsen, 2019). A recently developed nonwoven‐based gelatin membrane (NBM) is a new type of synthetic material produced by electrospinning technology from gelatin (Schulz et al., 2014). Gelatin has several advantages such as excellent biocompatibility, easy processing, low cost, and appears to be a promising candidate in clinical applications (Rose et al., 2014). NBM membranes are produced by electrospinning with in situ cross‐linking resulting in the formation of gradient fibrillary structures. These 3D structures closely mimic the native extracellular matrix (ECM) to promote cell growth and differentiation following the patterns similar to those found in native tissues and organs. NBM can be further modified by combining the gelatin gradient layer with the electrospun layer of polycaprolactone (PCL) and such biohybrid polymer membranes (BHM) possess superior mechanical properties in the aqueous environment (Angarano et al., 2013; Schulz et al., 2014).

One of the important requirements of regenerative biological material is the ability to stimulate the formation of new tissue and repair existing defects. On the cellular level, modern biological material should stimulate the migration of resident progenitor cells to the healing area, their proliferation, and differentiation into a mature tissue‐specific phenotype, as well as the production of new ECM (Boehler, Graham, & Shea, 2011; Nisbet, Forsythe, Shen, Finkelstein, & Horne, 2009; Skoog, Kumar, Narayan, & Goering, 2018). Some recent preclinical and histological studies with BHM have shown the unique physical and chemical properties and biological compatibility in vivo and in vitro (Jedrusik et al., 2018), but studies highlighting the potential to induce cellular processes and ECM synthesis compared with other commercial membranes on the market are incomplete. Gingival fibroblasts are the major resident progenitor cells of gingival tissue and play a crucial role in the regeneration of soft tissue defects (Andrukhov, Behm, Blufstein, & Rausch‐Fan, 2019; Smith, Martinez, Martinez, & McCulloch, 2019). Therefore, in the present study, we tested the biological behavior of commercially produced BHM on primary human gingival fibroblasts, expression of some differentiation markers as well as its capability to induce production of ECM. The biological response of gingival fibroblasts to BHM was compared with those to Mucograft, a 3D collagen matrix developed especially for soft tissue regeneration (Nevins, Nevins, Kim, Schupbach, & Kim, 2011).

## 2 MATERIALS AND METHODS

### 2.1 Specimen preparation

BHM was designed by Freiburg Materials Research Center and Institute for Macromolecular Chemistry of the Albert‐Ludwigs University Freiburg similar to the methods described previously (Strassburg et al., 2019). In the present study, commercially produced BHM (Neo Modulus [Suzhou] Medical, Jiangsu, China) was used. BHM and Mucograft® collagen matrix (MCM, Geistlich Biomaterials, Wolhusen, Switzerland) were cut into 6 mm diameter round with the help of a hole‐punch, and were sterilized by UV light for 45 min each side.

### 2.2 Cell culture

Human gingival fibroblasts (hGFs) were isolated from gingival tissue obtained from five periodontally healthy donors during routine extraction of their third molar teeth. The research protocol was approved by the Ethics Committee of the Medical University of Vienna. All donors were systematically healthy ranging in age from 22 to 28 years. All participants were informed in detail before the operation and gave their written consent. Cells were cultured in Dulbecco's modified Eagle's medium (DMEM) supplemented with 10% FBS (fetal bovine serum), streptomycin (50 μg/ml) and penicillin (100 U/ml) under humidified air atmosphere with 5% CO_2_ at 37°C. hGFs from passage levels 4–7 were used in the experiments.

### 2.3 Cell proliferation/viability

Cell proliferation/viability was measured using a cell counting kit (CCK‐8, Dojindo Laboratories, Japan) as previously described (Andrukhov et al., 2015). In these experiments, 1 × 10^4^ cells were seeded on either BHM or MCM (the porous layer facing up) membrane in 200 μl DMEM. Cells seeded at the same density on tissue culture plastic (TCP) were used as control. Cell proliferation/viability was measured after 3, 7, and 14 days of culture. Then, 20 μl of CCK‐8 reagent was added into each well and the culture plates were incubated in 5% CO_2_ at 37°C for 2 hr. Thereafter, 100 μl of each culture solution was transferred to a separate 96‐well plate and the optical density (OD) was measured at 450 nm using a microplate reader (Synergy HTX; BioTek) at 450 nm. The experiments were repeated five times with the cells isolated from five different donors.

Cell viability was further visualized using the LIVE/DEAD cell Staining Kit (Enzo Life Sciences AG, Lausen, Switzerland). Then, 1 × 10^4^ cells were seeded on either BHM or MCM (the porous layer faces up) membrane in 200 μl of DMEM. After 1, 3, and 7 days of culture, the cells were stained with 100 μl of staining solution at 37°C for 15 min and observed under a fluorescence microscope immediately after the staining.

### 2.4 Scanning electron microscopy analysis

The morphology and microstructure of hGFs grown on BHM and MCM were analyzed using scanning electron microscopy (SEM; Shi et al., 2017). hGFs were seeded on the BHM at a density of 1 × 10^4^ in 96‐well‐plate and cultured at 37°C as described above. Specimens in each group were scanned under SEM at 3, 7, and 14 days. For SEM, the specimens were fixed with 4% formaldehyde at least 24 hr and washed three times with PBS to remove unattached cells. Then, the specimens were dehydrated by rinsing with gradually increased ethanol. Afterward, ethanol was exchanged by hexamethyldisilazane (HMDS, Sigma‐Aldrich), the specimens were coated with gold and observed under the scanning electron microscope (SEM; JEOL‐JSM IT 300, JEOL, Tokyo, Japan) at an accelerating voltage of 15 kV. The SEM images of cross‐sectional and surface views were acquired.

### 2.5 Gene expression analysis

hGFs were seeded on the BHM at a density of 1 × 10^4^ in 96‐well‐plates in 200 µl DMEM. Cells seeded at similar density on TCP were used as control. After 3, 7, and 14 days of culture, the total mRNA was isolated using Cells‐to‐CT Bulk Lysis Reagents (Invitrogen, Carlsbad, CA) according to the manufacturer's instructions as previously described (Behm et al., 2019; Blufstein et al., 2019). mRNA samples were reversely transcribed into cDNA using the Cells‐to‐CT Bulk RT reagent (Ambion/Applied Biosystems, Foster City, CA). Quantitative real‐time PCR (qRT‐PCR) was performed in ABI StepOnePlus device (Applied Biosystems, Foster City, CA) using the Taqman gene expression assays (Applied Biosystems, Foster City, CA) with the following ID numbers: GAPDH, Hs99999905_m1; Type I collagen (Col1), Hs00164004_m1; Transforming growth factor β1 (TGF‐β1), Hs00998133_m1; Focal adhesion kinase (FAK), Hs00169444_m1; Fibroblast growth factor‐2 (FGF‐2), Hs00266645_m1; α‐smooth muscle actin (α‐SMA), Hs00909449_m1. qRT‐PCR reactions were performed in 96‐well plates using the following thermocycling conditions: 95°C for 10 min, 50 cycles, each for 15 s at 95°C and at 60°C for 60 s. The point at which the PCR product was first detected above a fixed threshold (termed cycle threshold, C_t_) was determined for each sample. Changes in the expression of target genes were calculated by a 2^−ΔΔCt^ method using the following formula: ΔΔ*C*_t_ = (*C*_t_^target^ − *C*_t_^GAPDH^)_sample_ − (*C*_t_^target^ − *C*_t_^GAPDH^)_control_.

### 2.6 Statistical analysis

The statistical differences between different groups were analyzed by one‐way analysis of ANOVA's statistic or Wilcoxon‐Test. All statistical analysis was performed using the statistic program SPSS 21.0 (SPSS, Chicago, IL). Data are expressed as means ± *SEM*. Differences were considered to be statistically significant at *p* < .05.

## 3 RESULTS

### 3.1 Proliferation/viability of hGFs grown on BHM and MCM

**Figure 1:**
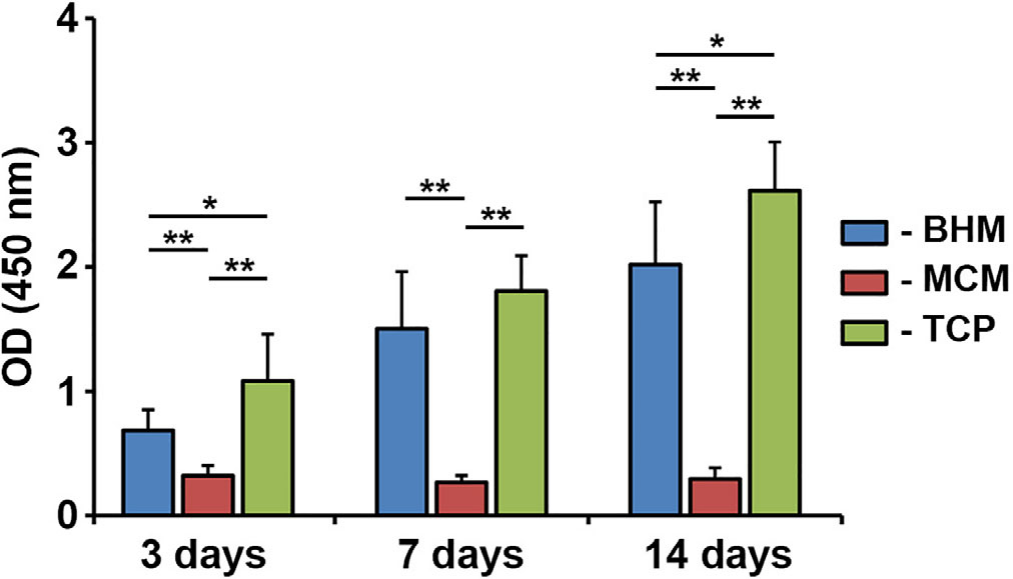
Proliferation/viability of primary human gingival fibroblasts grown on different membranes. Human gingival fibroblasts were seeded on biohybrid polymer (BHM), Mucograft® collagen matrix (MCM), and TCP and their proliferation/viability were tested using CCK‐8 test at 3, 7, and 14 days. Data are presented as means ± *SD* of OD values (450 nm) measured in five independent experiments with cells of five different donors. One‐way ANOVA (*n* = 5), Difference between groups are indicated by: *p < .05, ** *p* < .01

Proliferation/viability of primary hGFs grown on BHM, MCM, and TCP group after different culture times are summarized in Figure 1. At all investigated time points (3, 7, and 14 days), proliferation/viability of hGFs grown on MCM was significantly lower compared to those grown on BHM and TCP (*p* < .05). No statistically significant difference in proliferation/viability was observed between hGFs grown on BHM and TCP (*p* > .05). Proliferation/viability of hGFs grown on BHM and TCP was gradually increased with prolonged culture time. In contrast, hGFs grown on MCM exhibited similar OD values after all observation time points suggesting no proliferation.

**Figure 2:**
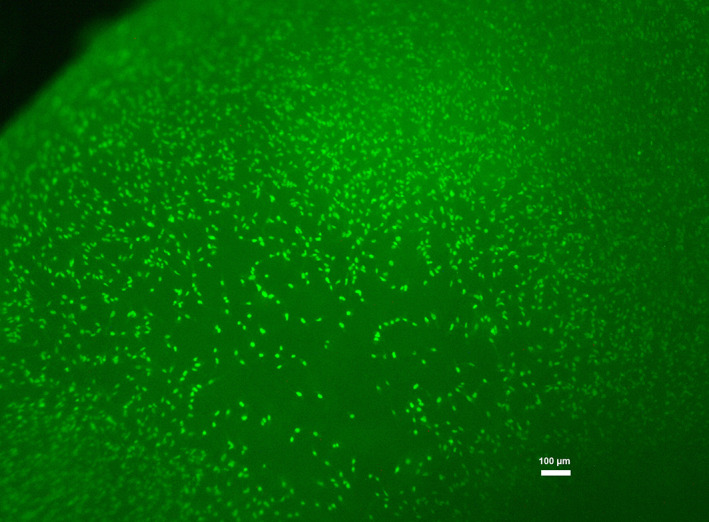
Live‐dead staining of human gingival fibroblasts grown on BHM for 7 days

Live/dead staining of hGFs grown on showed that most cells were viable, dead cells were not observed (Figure 2).

### 3.2 SEM analysis

**Figure 3:**
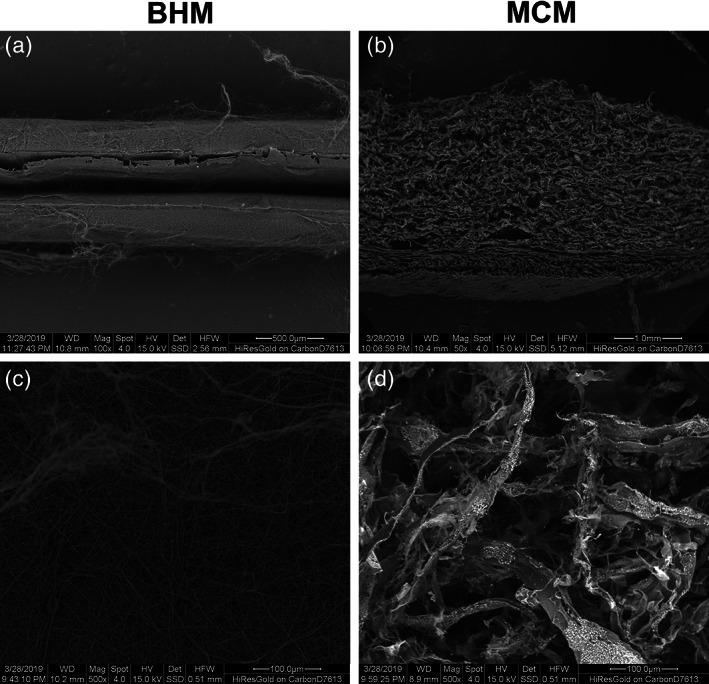
SEM analysis of BHM and MCM. Microscopic structural characteristics of MCM and BHM observed at different projections. Panels (a) and (b) show the cross‐sections of the analyzed materials (a = BHM) and (b = MCM) and panels (c) and (d) show the spongy surface of BHM and MCM surface separately. Scale bar is given for each picture

SEM images showing structural features of two membranes are shown in Figure 3. Although MCM and BHM are both multilayered structures, obvious differences were detected between them. The outer two layers of BHM are gelatin prepared by electrospinning, and the middle layer is PCL layer (Figure 3a). MCM is composed of a compact layer and a spongy layer (Figure 3b). Compared to BHM, the double‐layer matrix of MCM is markedly thicker and upholds a nearly three‐fold volume. The BHM surface is smoother than the spongy layer of MCM. The pore size of BHM surface is visibly smaller than that of MCM (Figure 3c,d).

**Figure 4:**
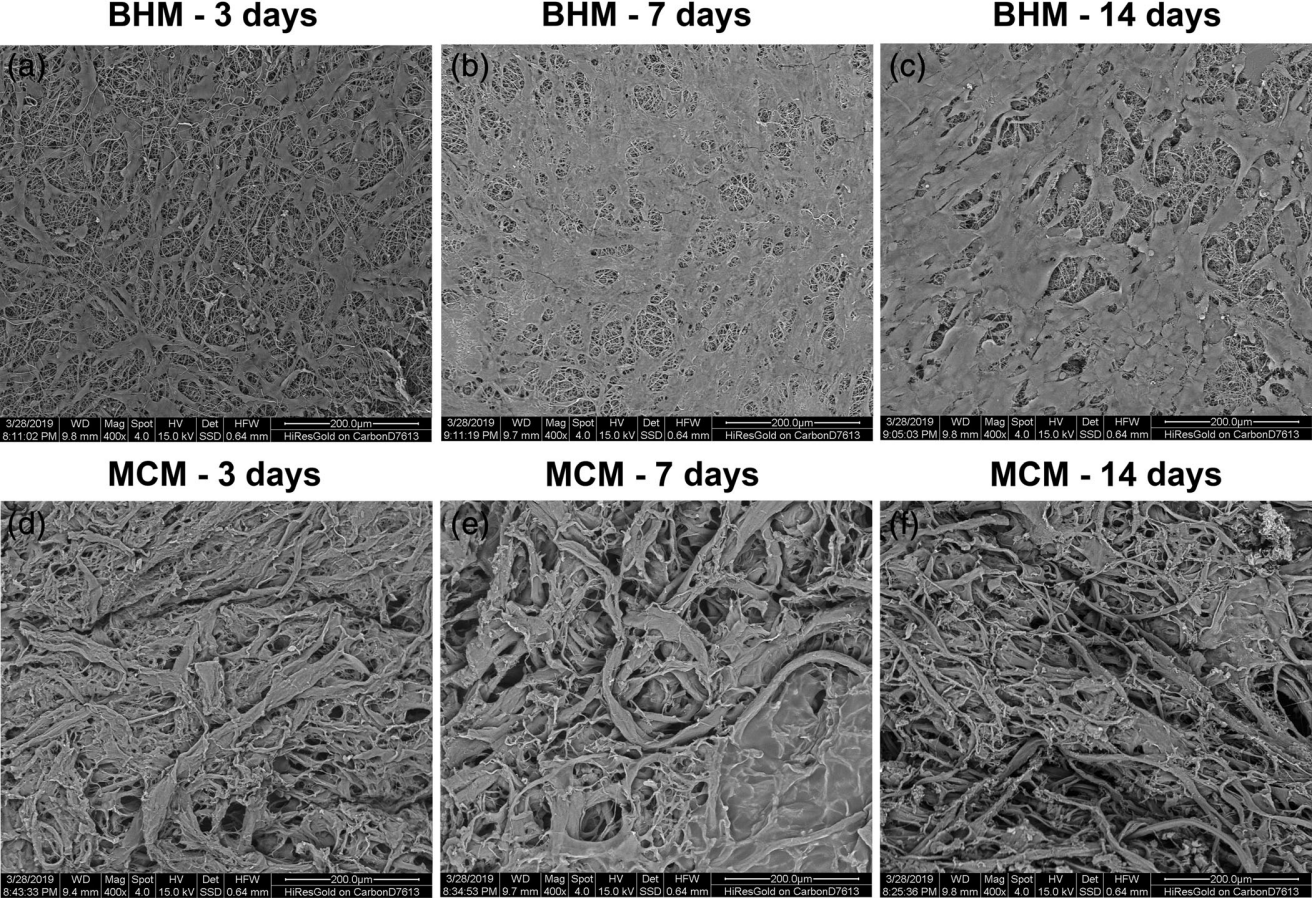
Scanning electron microscopy analysis of hGFs grown on BHM and MCM. HGFs after (a, d) 3, (b, e) 7, and (c, f) 14 days of culture. Scale bar = 200 μm

Representative SEM pictures of hGFs grown on BHM and MCM after different periods are presented in Figure 4. On BHM, after 3 days hGFs were attached to the membrane and exhibited typical fibroblast morphology (Figure 4a). After 7 days of culture, the cells were nearly confluent and covered almost the whole membrane surface (Figure 4b). After 14 days, some multilayered cell structures could be observed (Figure 4c). On MCM, cells were hardly observed throughout the whole observation period (Figure 4d–f).

### 3.3 Gene expression analysis

**Figure 5:**
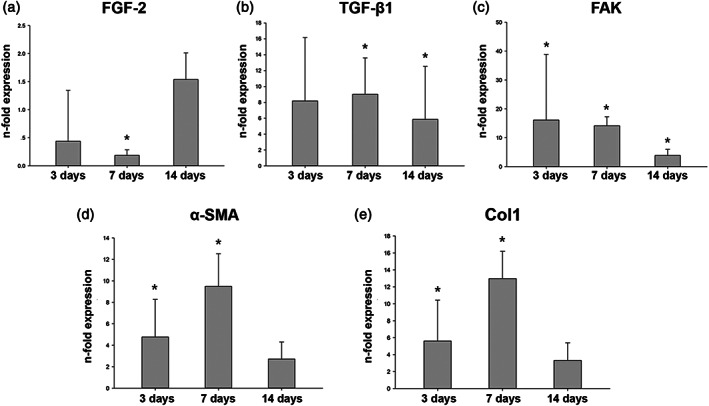
Gene expression analysis of hGFs grown on BHM. Expression of FGF‐2, TGF‐β1, FAK, Col1, α‐SMA in hGFs grown on BHM at 3, 7, and 14 days: (a) FGF‐2; (b) TGF‐β1; (c) FAK; (d) Col1; (e) α‐SMA. *Y*‐axis represents the *n*‐fold expression levels of the target gene in relation to cells in TCP group (control) calculated by 2^−ΔΔCt^ method. Data are presented as mean ± *SEM* of five independent experiments with hGFs of five different donors (*n* = 5), *significant difference between groups as tested by Wilcoxon test (*p* < .05)

Since MCM did not support the growth of HGFs, gene expression analysis was limited only to BHM group. The expression of several genes in hGFs grown on BHM in comparison with TCP after 3, 7, and 14 days of culture is shown in Figure 5. We focused on the expression of FGF‐2, TGF‐β1, FAK, Col1, and α‐SMA, which are involved in the process of soft tissue formation. The expression of FGF‐2 in BHM group was lower compared to TCP group after 7 days (*p* < .05, Figure 5a), but after 14 days FGF‐2 expression was similar in both groups. The expression of other investigated genes was generally higher in hGFs grown on BHM compared to those grown on TCP at all time points. Significant differences were observed for TGF‐β1 after 7 and 14 days (*p* < .01, Figure 5b), for FAK at all time points (*p* < .01, Figure 5c), for α‐SMA and Col1 after 3 and 7 days (*p* < .01, Figure 5d, e).

## 4 DISCUSSION

The purpose of this study was to evaluate the in vitro biocompatibility of BHM in hGFs and the capability to support cell adhesion and proliferation as well as the ability to influence the expression of some factors involved in gingival tissue regeneration and ECM production. hGFs are the most common gingival progenitor cells and play an important role in repairing tissue damage and maintain periodontal health (Andrukhov et al., 2019; Smith et al., 2019). Modern biomaterials designed for periodontal soft tissue regeneration are expected to stimulate the adhesion, migration, and proliferation of hGFs as well as production of specific factors involved in the processes of gingival connective tissue formation and remodeling (Cao, Wang, Pu, Tang, & Meng, 2018). Physical and mechanical features of scaffold such as material, topography, rigidity, and porosity substantially influence different cellular characteristics and subsequently the regenerative processes (James, Levene, Parsons, & Kohn, 1999; Stevens & George, 2005; Yeung et al., 2005). Therefore, specific modifications of scaffold physicochemical properties might be useful for the achievement of the regeneration of highly specific gingival soft tissue through stimulation of the resident progenitor cells such as gingival fibroblasts.

In our study, we compared the surface characteristics of BHM and MCM and their effects on the functional properties of primary hGFs. BHM is a newly developed membrane composed of gelatin and polycaprolactone. On the one hand, it serves as a barrier membrane but on the other hand, its surface has a 3D structures composed of cross‐linked nanofibers serving as scaffold (Jedrusik et al., 2018). MCM is one of the types of resorbing porcine xenogeneic materials, which is a pure collagen type I and type III matrix obtained with a standardized, controlled manufacturing process without any cross‐linking or chemical treatment. It consists of two functional layers: one thin, smooth, low‐porosity compact layer and one thicker, porous, 3D spongy layer (Ghanaati et al., 2011). The matrix is a resorbable 3D matrix designed specifically for soft tissue regeneration in the oral cavity and for the replacement of autologous grafts (Carter et al., 2017; Menceva et al., 2018; Ramachandra, Rana, Reetika, & Jithendra, 2014; Schmitt & Moest, 2016). In clinical application, the compact layer contacts the epithelial cells outward, and the porous layer contacts the fibroblasts inward, so we choose the spongy layer of MCM membrane as the surface for cell culture.

The results of CCK‐8 showed that the cell viability/proliferation of hGFs grown on BHM and TCP was gradually increased up to 14 days of culture. As can be seen on the SEM images, the density of cells growing on BHM was gradually decreased until the membrane was fully covered with hGFs monolayer. Live/Dead staining showed that BHM had no cytotoxic effect on the hGFs during the whole culture period. Thus, we can conclude that BHM shows excellent biocompatibility and supports the proliferation of primary hGFs. These observations are in agreement with previous reports, showing that nonwoven‐based gelatin membranes support attachment and viability of different cell types, and particularly hGFs (Schulz et al., 2014; Strassburg et al., 2019).

In contrast, the viability/proliferation of cells on MCM was not changed during 14 days. Surprisingly, we observed that collagen‐based Mucograft membrane does not support the proliferation of primary hGFs. This observation seems to contradict to a former study showing a gradual proliferation of hGFs in the presence of MCM (Lima et al., 2015). The reasons for the contradictory observations could be some differences in the protocols between these studies. In our study, the pieces of membranes fit the dimensions of the cell culture well, so that that seeded hGFs grew mainly on membranes and not on TCP. In contrast, the size of the membrane used in a previous study was <50% of the well diameter (Lima et al., 2015), suggesting that the cells grew without direct contact with the membrane. Although in our study hGFs did not grow on MCM membrane, clinical studies suggest its positive effect on soft tissue regeneration (Carter et al., 2017; Menceva et al., 2018; Ramachandra et al., 2014; Schmitt & Moest, 2016). Therefore, we can conclude that the biological effects of MCM are explained by other mechanisms that stimulate progenitor cells proliferation.

Ideal bioresorbable material for oral surgery should provide not only cell attachment and proliferation, but also influence the synthesis, transport, and secretion of extracellular matrix protein and growth factors. Immediately after surgical procedure, resident progenitor cells are attracted by various clot‐derived factors into the wound area, where they adhere to temporary scaffold and start to proliferate. HGFs start to produce different growth factors and ECM proteins required for new tissue formation. The process of new tissue formation is complicated and largely determined by the continuous dynamic interaction of HFGs with ECM. ECM influences the behavior and activity of HGFs, which secrete new proteins participating in ECM formation and remodeling. The contacts of cells with ECM are mediated by focal adhesion (FA) complex, which is an assembly of different proteins involved in the adhesion to ECM and subsequent activation of various intracellular signaling pathways FAK, a nonreceptor protein tyrosine kinase, localizes to integrin‐rich cellular FA sites (Fischer, Wong, Baruth, & Cerutis, 2017). The autophosphorylation of FAK is critical for the regulation of adhesion enhancement (Michael, Dumbauld, Burns, Hanks, & Garcia, 2009). We have observed that FAK expression in hGFs on BHM was significantly increased compared to TCP at the initial phase of culture, which can be explained by the fact that BHM is a scaffold with a 3D structure. This assumption is in line with a former observation that the expression of the proteins implicated in the FA in gingival fibroblast grown on 3D gelatin nanofibrous scaffold is significantly increased compared to the 2D plastic surface (Sachar et al., 2014). One recent study implies that hGFs exhibited lower expression of FAK compared to dermal fibroblasts, and this was associated with the decreased spreading and adhesion ability (Guo, Carter, Mukhopadhyay, & Leask, 2011). Thus, our data suggest that BHM membrane due to its structural and material characteristics support adherence of hGFs. The expression of FAK in hGFs on BHM was decreased after 14 days. At this time point, cells that grew on BHM already reached confluence and started to form some multilayered structures. A decreased direct contact of hGFs with BHM after prolonged culture and confluence could be associated with a decreased FAK expression.

Previous studies have shown that elevated FAK expression induces the expression of α‐SMA, which plays an important role in mechanotransduction and tissue remodeling (Guo, Carter, & Leask, 2014). Furthermore, the expression of Col1, which is the most important component of the ECM, is also be enhanced by FAK (Cheung, McCulloch, & Santerre, 2014). The expression of both α‐SMA and Col1 in hGFs grown on BHM was substantially increased after 3 and 7 days, which could be associated with an increased FAK expression. High expression of α‐SMA and Col1 suggests an increased production of new ECM and improved ECM remodeling. After 14 days, when FAK expression was decreased, we did not observe any significant differences in α‐SMA and Col1 expression between BHM and TCP. This observation supports our assumption about the association between FAK expression on the one hand and α‐SMA and Col1 expression on the other hand.

The other important read‐outs of our study were TGF‐β1 and a member of fibroblast growth factors family protein FGF‐2. These growth factors play an important role in the regeneration of periodontal tissue, particularly bone defect (Kitamura et al., 2011; Maeda, Wada, Tomokiyo, Monnouchi, & Akamine, 2013; Ripamonti, 2019). Interestingly, these growth factors were differently regulated by in hGFs grown on BHM. The expression of TGF‐β1 in BHM group was higher than that in TCP group over the whole observation period. Previous studies show that TGF‐β1 might induce the expression of α‐SMA (Murphy‐Marshman et al., 2017) and Col1 (Chen et al., 1999) through the Smad signaling pathway. Therefore, increased expression of α‐SMA and Col1 could be partially explained by TGF‐β1 dependent mechanisms. Our data are also in agreement with a recent study showing that hGFs cultured on polycaprolactone/gelatin nanopolymers scaffold with incorporated 2‐hydroxy‐1,4‐naphthoquinone exhibit an increased expression of TGF‐β1 and Col1 (Adeli‐Sardou, Yaghoobi, Torkzadeh‐Mahani, & Dodel, 2019).

Surprisingly, the expression of FGF‐2 was decreased in the GFs grown on BHM compared to those grown on TCP after 3 and 7 days of culture but recovered after 14 days of culture. The reason for this could be a reciprocal relationship between FGF‐2 signaling and proliferation. We can assume that FGF‐2 expression can play a more important role in the later phases of the regenerative process such as tissue maturation and remodeling. Therefore, decreased expression of FGF‐2 during the first proliferative phase could be physiologically important, but this question needs to be further explored. One study shows that at higher amounts, FGF‐2 might inhibit the proliferation of hGFs (Ma et al., 2012). Besides, there is a reciprocal relationship between FGF‐2 and TGF‐β1 signaling (Liguori, Liguori, Moreira, & Harmsen, 2018). Furthermore, FGF‐2 decreases the expression of α‐SMA and inhibits myofibroblast differentiation (Akasaka et al., 2007, 2010), which is in agreement with the expression pattern observed in our study.

There are still some limitations in our experiments. First, we only chose HGFs as experimental cells, but the gingival soft tissue is a mixture of cells. As shown by recent reports, gelatin‐based membrane affects epithelial cells as well as their communication with gingival fibroblasts (Jedrusik et al., 2019; Strassburg et al., 2019). Second, we could not imitate the complex oral microenvironment in vitro study. Therefore, when trying to translate our results to finally clinical application, it should be quite cautious and further studies are still necessary.

## 5 CONCLUSIONS

In summary, BHM stimulates HGFs adhesion, migration, and differentiation in vitro. It decreases the gene expression of FGF‐2, increases the gene expression of FAK and TGF‐β1, then enhances the gene expression Col1 and α‐SMA. These data provide the first scientific evidence to support the BHM as a potential material could be used in soft tissue augmentation.
